# Identification of Quantitative Trait Loci Associated With Iron Deficiency Tolerance in Maize

**DOI:** 10.3389/fpls.2022.805247

**Published:** 2022-04-14

**Authors:** Jianqin Xu, Xiaoyang Zhu, Fang Yan, Huaqing Zhu, Xiuyu Zhou, Futong Yu

**Affiliations:** ^1^Key Laboratory of Plant-Soil Interaction (MOE), Centre for Resources, Environment and Food Security, College of Resources and Environmental Sciences, China Agricultural University, Beijing, China; ^2^Key Lab of Crop Heterosis and Utilization of Ministry of Education, Beijing Key Lab of Crop Genetic Improvement, China Agricultural University, Beijing, China

**Keywords:** maize (*Zea mays* L.), iron (Fe) deficiency tolerance, iron efficiency, nitrogen form, quantitative trait locus (QTL)

## Abstract

Iron (Fe) is a limiting factor in crop growth and nutritional quality because of its low solubility. However, the current understanding of how major crops respond to Fe deficiency and the genetic basis remains limited. In the present study, Fe-efficient inbred line Ye478 and Fe-inefficient inbred line Wu312 and their recombinant inbred line (RIL) population were utilized to reveal the physiological and genetic responses of maize to low Fe stress. Compared with the Fe-sufficient conditions (+Fe: 200 μM), Fe-deficient supply (−Fe: 30 μM) significantly reduced shoot and root dry weights, leaf SPAD of Fe-efficient inbred line Ye478 by 31.4, 31.8, and 46.0%, respectively; decreased Fe-inefficient inbred line Wu312 by 72.0, 45.1, and 84.1%, respectively. Under Fe deficiency, compared with the supply of calcium nitrate (N1), supplying ammonium nitrate (N2) significantly increased the shoot and root dry weights of Wu312 by 37.5 and 51.6%, respectively; and enhanced Ye478 by 23.9 and 45.1%, respectively. Compared with N1, N2 resulted in a 70.0% decrease of the root Fe concentration for Wu312 in the −Fe treatment, N2 treatment reduced the root Fe concentration of Ye478 by 55.8% in the −Fe treatment. These findings indicated that, compared with only supplying nitrate nitrogen, combined supply of ammonium nitrogen and nitrate nitrogen not only contributed to better growth in maize but also significantly reduced Fe concentration in roots. In linkage analysis, ten quantitative trait loci (QTLs) associated with Fe deficiency tolerance were detected, explaining 6.2–12.0% of phenotypic variation. Candidate genes considered to be associated with the mechanisms underlying Fe deficiency tolerance were identified within a single locus or QTL co-localization, including *ZmYS3*, *ZmPYE*, *ZmEIL3*, *ZmMYB153*, *ZmILR3* and *ZmNAS4*, which may form a sophisticated network to regulate the uptake, transport and redistribution of Fe. Furthermore, *ZmYS3* was highly induced by Fe deficiency in the roots; *ZmPYE* and *ZmEIL3*, which may be involved in Fe homeostasis in strategy I plants, were significantly upregulated in the shoots and roots under low Fe stress; *ZmMYB153* was Fe-deficiency inducible in the shoots. Our findings will provide a comprehensive insight into the physiological and genetic basis of Fe deficiency tolerance.

## Introduction

Iron (Fe) is an essential micronutrient with numerous cellular functions in animals and plants (Zhou et al., 2013). About 80% of the cellular Fe in plant leaves is located in the chloroplasts, which is the vital pigment required for photosynthesis (Zhang et al., 2017; Long et al., 2020). Also, the evolutionary ability of Fe to change oxidation states between Fe(III) and Fe(II) renders it irreplaceably important in many essential processes associated with basic redox reactions (Li and Lan, 2015). However, Fe is found in nature mostly as insoluble Fe(III) oxyhydroxides that are sparingly soluble in aerobic, neutral pH soils because of many adverse soil properties (Tognetti et al., 2007; Moreno-Jiménez et al., 2019; Housh et al., 2021). Fe deficiency can significantly reduce crop yield and quality, which, in turn, affects the immune and nervous systems, the mental and intellectual development of humans who consume these crops (Li et al., 2019). Fe cannot be synthesized by humans and must be acquired through the diet (Hindu et al., 2018). In developing countries that rely on cereal diets, for example, in China, more than 300 million people are at risk of Fe and zinc (Zn) deficiencies (Zhao et al., 2020). Additionally, inadequate intake of Fe results in Fe-deficiency anemia (IDA), affecting more than half of the world’s population (Wang et al., 2019). In the worst-case scenario, Fe deficiency may cause human death (Prasad, 2013; WHO, 2013).

Maize is the world’s most-produced food crop and accounts for 41% of the world’s total grain production (Tian et al., 2019). And maize provides 15 and 20% of the total proteins and calories, respectively, consumed by the world’s population (Cheah et al., 2019; Guo et al., 2020). And maize ranks as the top cereal crop in terms of total production and planting area in China (Food and Agriculture Organization of the United Nations [FAO], 2018). Fe deficiency considerably restricts maize production, hence threatening food security. It supplies not only energy in the form of carbohydrates, fat, and proteins but also vitamins and many minerals, including Fe (Wang et al., 2019). Meanwhile, maize is not only a major crop plant for the feed industry and food, but also a model plant for genetics and evolutionary study (Zhou et al., 2013). Maize is considered a graminaceous species with low phytosiderophore release, and little is known about other determinants contributing to its tolerance to Fe deficiency-induced chlorosis (Shi et al., 2018). Investigating the mechanisms of Fe acquisition, translocation, and homeostasis in maize may support a model for understanding that in other crops and provide gene resources for further breeding maize varieties with enhanced Fe content.

Moreover, Fe deficiency in humans is a global health issue, affecting about 25% of the world’s population (1.62 billion) (Chan-Rodriguez and Walker, 2018). At present, information on the molecular mechanisms of Fe absorption, translocation, and accumulation in maize under low Fe stress is still limited. The main reason is that there are few works to identify related genes by using methods such as population genetics approach. These works are of great significance for maintaining Fe homeostasis and bio-enhancement in plants under Fe starvation. Under low Fe stress, plants have evolved two distinct strategies to solubilize and transport Fe, such as the reduction-based strategy (Strategy I) in dicotyledonous and non-graminaceous monocotyledonous plants, and the chelation-based strategy (Strategy II) for graminaceous monocots (Li et al., 2014; Kurt et al., 2019). The characteristic of strategy I is the release of a proton (H^+^) from the roots (mediated by AHA2), which facilitates the mobilization and subsequent reduction of Fe(III) to Fe(II) *via* the actions of plasma membrane-bound Fe(III), chelate reductase (FRO2) (Guerinot, 2007; Benke et al., 2015; Li and Lan, 2015). The soluble Fe(II) is finally transported into the root epidermis by the divalent metal transporter (iron-regulated Transporter 1, IRT1) (Vert et al., 2002; Mallikarjuna et al., 2020). Strategy II plants, like maize, secrete mugineic acids (MAs; also known as phytosiderophores) into the rhizosphere using efflux transporters (TOM1 and TOM2) (Nozoye et al., 2011). The MAs chelate inorganic Fe(III) in the soil, producing Fe(III)-MAs complexes that are subsequently absorbed through the specific transporter yellow stripe 1 (YS1) (Benke et al., 2015).

Transcriptomic analysis of Fe deficiency response reveals that a strong modulation of genes involved in regulatory aspects, Fe translocation, root morphological modification, primary metabolic pathways, and hormonal metabolism are induced by the Fe nutritional stress (Zanin et al., 2017). Fe-deficiency stress is signaled by many plant hormones, including indole acetic acid (IAA), ethylene, abscisic acid (ABA), gibberellins, cytokinins, and brassinosteroids (Ivanov et al., 2012; Wang et al., 2012). The interaction between the auxin and NO modulates the root growth under Fe deficiency in rice (Sun et al., 2017). Also, ethylene regulates *FRO2* and *IRT1* gene expression through the modulation of the major transcription factor FER or FIT (FER-like iron deficiency-induced transportation factor) (Ye et al., 2015). The FIT forms heterodimers with bHLH38 and bHLH39, and positively regulates a subset of Fe-responsive genes (Yuan et al., 2008). In addition, bHLH115 proteins have bZIP, bHLH, and MYB transcription factor-binding sites, which strengthen their engagement in various metabolic ways (Li et al., 2016). The POPEYE (PYE) plays a negative role in the Fe deficiency response in *Arabidopsis* by repressing the expression of *nicotianamine synthase 4* (*NAS4*), *FRO3*, and *zinc-induced facilitator1* (*ZIF1*) (Kurt et al., 2019). And, in *Arabidopsis*, the mitogen-activated protein kinase 3 and 6 (MPK3/MPK6)-regulated ACS2/6 activation is part of the Fe deficiency-induced ethylene production-signaling pathway (Ye et al., 2015). These Fe deficiency-inducible genes are regulated at transcriptional, translational, and post-translational levels. However, the functions of these genes are mainly verified in the strategy I plants.

Few genes related to Fe homeostasis have been studied in maize. *ZmYS1* expression at both the messenger RNA (mRNA) and protein levels responds rapidly to changes in Fe availability (Chorianopoulou et al., 2015). ZmNAS proteins of Class I are mostly responsible for the long-distance translocation of Fe in stems, and their expression can be expanded in the epidermis, shoot apices as a response to Fe starvation (Zhou et al., 2013). The *ZmZIP5* may play a role in Zn and Fe uptake and root-to-shoot translocation (Li et al., 2019). Fe concentrations are elevated in the shoots and roots of ectopic *ZmZIP7*-overexpressing plants, while the endogenic Fe deficiency-inducible genes are stimulated in transgenic *Arabidopsis* (Li et al., 2016). The ZmFDR4 functions as a Fe transporter in monocot plastids, which subsequently increases chlorophyll content and enhances photosynthetic efficiency, thus, affects plastid development (Zhang et al., 2017). Genes related to Fe homeostasis could be employed to fortify the Fe contents in plants, thus boosting micronutrient availability for human populations, depending on staple crops (Ricachenevsky et al., 2018; Li et al., 2019). Therefore, identifying and analyzing more genes related to low Fe stress tolerance in maize may play an important role in the growth and development of maize and human health.

In the current study, several supply levels of Fe(III)-EDTA were designed to evaluate more reasonable and effective indicators to characterize Fe deficiency tolerance between the Fe-inefficient (Wu312) and Fe-efficient (Ye478) inbred lines at the seedling stage in Experiment 1, which allowed for linkage analysis in the RIL population in Experiment 3. Under low Fe stress, there is still very limited information on the differences in the response of Fe-inefficient and Fe-efficient maize inbred lines to different nitrogen supply forms. Also, so far, mainly supplying nitrate nitrogen, which is recommended to use in the Hoagland nutrient solution, has significantly affected the accuracy for measuring Fe concentration in roots. According to the results in Experiment 1, Experiment 2 was designed to analyze the effects of different nitrogen supply forms on the plant growth, root Fe concentration under Fe-deficient and Fe-sufficient conditions. Furthermore, the recombinant inbred line (RIL) population derived from the Fe-inefficient (Wu312) and Fe-efficient (Ye478) inbred lines were utilized to identify the quantitative trait loci and candidate genes associated with Fe efficiency in maize. The expression of candidate genes probably involved in the mechanisms underlying Fe deficiency tolerance in maize was assessed in the shoots and roots of Fe-inefficient (Wu312) and Fe-efficient (Ye478) parents under different Fe nutritional statuses.

## Materials and Methods

### Plant Material and Experimental Design

**FIGURE 1:**
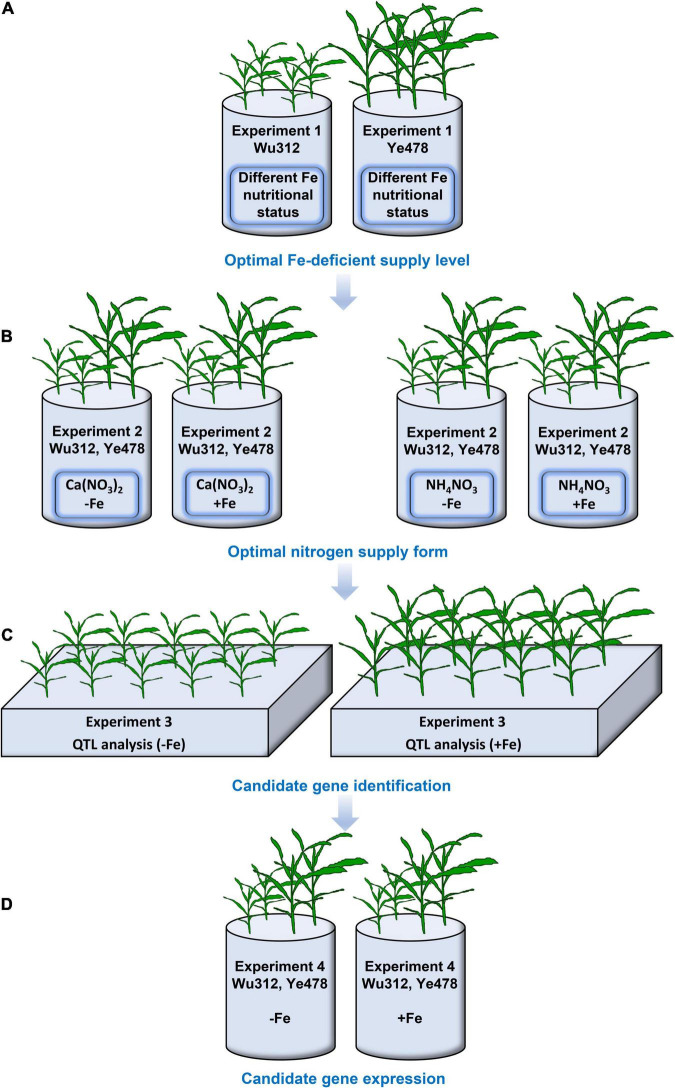
An experimental setup in this study. **(A)** In Experiment 1, eight different Fe(III)-EDTA levels (0, 10, 30, 50, 70, 100, 200, and 300 μM) were designed. And four seedlings of inbred lines Wu312 or Ye478 were hydroponically grown in a 3.3-L container as a replicate. Each treatment contained three replicates. **(B)** In Experiment 2, two seedlings of each inbred line were grown in a 3.3-L container supplied with Ca (NO_3_)_2_ [4 mM NO_3_^–^-N] or NH_4_NO_3_ [2-mM NO_3_^–^-N and 2-mM NH_4_^+^-N] under Fe-deficient [–Fe: 30-μM Fe(III)-EDTA] and Fe-sufficient conditions [+Fe: 200-μM Fe(III)-EDTA] as a replicate. Each treatment contained three replicates. **(C)** In Experiment 3, a recombinant inbred line (RIL) population derived from Fe-inefficient inbred line Wu312 and Fe-efficient inbred line Ye478 was grown under the –Fe and +Fe conditions to identify the quantitative trait loci associated with Fe deficiency tolerance. Each treatment contained three replicates. **(D)** In Experiment 4, the expression of candidate genes identified from Experiment 3 was analyzed in the shoots and roots of Wu312 and Ye478 under the –Fe and +Fe conditions. Each treatment contained three replicates.

In Experiment 1, eight different Fe treatments were designed, containing 0, 10, 30, 50, 70, 100, 200, and 300 μM Fe(III)-EDTA. Two seedings of Wu312 and Ye478 were grown in a 3.3-L container under different Fe nutritional statuses in the same growth chamber (Figure 1A) as a replicate. In Experiment 2, two seedlings for each inbred line were grown in a 3.3-L container supplied with Ca (NO_3_)_2_ [4 mM NO_3_^–^-N] or NH_4_NO_3_ [2 mM NO_3_^–^-N, 2 mM NH_4_^+^-N] under Fe-deficient [−Fe: 30 μM Fe(III)-EDTA] and Fe-sufficient conditions [+Fe: 200 μM Fe(III)-EDTA] as a replicate (Figure 1B). Each treatment contained three replicates, and a total of six seedlings for each inbred line were used for each treatment in Experiment 2.

In Experiment 3, the Ye478 × Wu312 RIL population, which consists of 218 lines, was derived from Ye478 (a female parent) and Wu312 (a male parent) as described by Liu et al. (2011), and their parents were hydroponically grown under Fe-deficient [−Fe: 30 μM Fe(III)-EDTA] and Fe-sufficient conditions [+Fe: 200 μM Fe(III)-EDTA] (Figure 1C). Each treatment contained three replicates.

In Experiment 4, the expression of candidate genes associated with Fe deficiency tolerance identified from Experiment 3 was analyzed in the shoots and roots of Fe-inefficient (Wu312), and Fe-efficient (Ye478) parents under Fe-deficient [−Fe: 30 μM Fe(III)-EDTA] and Fe-sufficient [+Fe: 200 μM Fe(III)-EDTA] conditions (Figure 1D).

### Plant Growth and Hydroponic Culture

Maize seeds were sterilized for 30 min in a 10% solution of H_2_O_2_, washed with distilled water, soaked in saturated CaSO_4_ for 10 h, and then germinated on moist filter paper in the dark at room temperature. Two days later, the germinated seeds were wrapped in a moist filter paper roll and grown. At the stage of two visible leaves, the seedlings were selected and transferred into a 3.3-L container (Experiments 1, 2 and 4) or a 40-L container (Experiment 3). The adjusted Hoagland nutrient solution contained (mM) 0.75 K_2_SO_4_, 0.65 MgSO_4_, 0.1 KCl, 0.25 KH_2_PO_4_, 1.0 × 10^–3^ H_3_BO_3_, 1 × 10^–2^ ZnSO_4_, 1 × 10^–4^ CuSO_4_, 1 × 10^–3^ MnSO_4_, 5 × 10^–6^ (NH_4_) Mo_7_O_24_, 1 × 10^–4^ NiCl. Solution pH was set at 5.5–6. The nutrient solution was renewed every 3 days and aerated by a pump. The growth chamber condition was set as a 14-h light period from 8:00 to 22:00 with 28°C and a 10-h-dark period with 22°C. The average light intensity measured at a canopy was 350 μmol m^–2^ s^–1^.

### Data Collection

During the period from 9:00 to 11:00 a.m. on the 14th day after transfer; the SPAD value of the fourth leaf was measured on the 1/3 parts from the leaf base three times using a SPAD-502 leaf chlorophyll meter. The average of three observed values was recorded for each plant. Plants of all experiments were harvested on the 14th day after transfer. Plant height and root length for each plant were recorded. In Experiment 1, active Fe concentration was determined in young leaves and old leaves for each plant. About 2 g fresh samples were soaked in 20 ml 1 M HCl solution and shaken for 5 h at 25°C. Then, the solution was filtered, and Fe concentration was measured by ICP-AES (Inductively Coupled Plasma-Atomic Emission Spectroscopy). Active Fe concentration was expressed as μg g^–1^ fresh weight (FW). The roots in Experiment 1 were only washed by distilled water. The roots in Experiment 2 were washed using a mixed solution of EDTA-Na and CaSO_4_, subsequently washed by 1 M HCl solution, and finally washed using distilled water. Then, the shoot and root for each plant were separately collected in an envelope. All samples were heat-treated at 105°C for 30 min and dried at 75°C until constant weight. Subsequently, Fe concentrations in shoots and roots were measured by ICP-AES. Fe efficiency based on shoot (root) dry weight, Fe content, Fe uptake efficiency, and Fe transport efficiency was estimated using the following equations from (1) to (4), respectively.

(1) Fe efficiency based on shoot (root) dry weight (%) = shoot (root) dry weight under Fe-deficient conditions × 100%/shoot (root) dry weight under Fe-sufficient conditions.
(2) Fe content (μg plant^–1^) = concentration × dry weight.
(3) Fe uptake efficiency (μg root dry weight g^–1^) = (shoot Fe content + root Fe content)/root dry weight.
(4) Fe transport efficiency (%) = shoot Fe content × 100%/(shoot Fe content + root Fe content).

### Statistical Analysis

The mean for each trait was compared using one-way ANOVA at a 0.05 level of probability followed by the least significant difference (LSD) test. Means between inbred lines Wu312 and Ye478 in each experiment and the means of each parent between the −Fe and +Fe treatments in Experiment 3 were compared using Student’s *t*-test. In Experiment 3, the linear mixed effect function lmer from the lme4 package in R was fitted to each RIL to obtain the best linear unbiased prediction (BLUP) value for each trait. Broad-sense heritability for each trait was calculated using *H*^2^ = σ_*g*_^2^/(σ_*g*_^2^ + σ_*ge*_^2^/*e* + σ_ε_^2^/*re*), where σ_*g*_^2^ is genetic variance, σ_*ge*_^2^ is the interaction of genotype and treatment, σ_ε_^2^ is a residual error, while *e* and *r* are the number of environments and replicates, respectively. The Kolmogorov-Smirnova test was performed as the statistical method to verify the normality in the distribution for each trait in the RIL population.

### Quantitative Trait Locus Analysis

Quantitative trait locus (QTL) analysis was performed using composite interval mapping (CIM) in the Windows QTL Cartographer version 2.5. Model 6 was selected for detecting QTL and estimating their effects. The threshold logarithm of odds (LOD) values to declare the putative QTL was estimated by permutation tests with a minimum of 1,000 replicates at a significant level of *p* < 0.05 (LOD = 2.9). The confidence interval for each QTL was determined using the 1-LOD interval method, which was calculated and defined by left and right markers. The QTLs for the same trait having the same confidence intervals were defined as identical QTL.

### Annotation of Candidate Genes

According to the physical distance, genes within the refined interval and their functional descriptions were identified using the maize B73 reference genome assembly available on the MaizeGDB database^1^ and Gramene Database^2^.

### RNA Extraction and Gene Expression Quantification

In Experiment 4, the total RNA was extracted from shoots and roots of plants using the Total RNA Extraction Kit (TIANGEN, China). The complementary DNA (cDNA) was synthesized in accordance with Fast Quant RT Super MixReverse Transcription Kit instructions (TransGene, Beijing, China). Quantitative real-time PCR was performed using SYBR Green Real-time RT-PCR (Takara) and an ABI7500 Fast Real-Time PCR System (Applied Biosystems). The primers used for real-time PCR are shown in Supplementary Table 1. The relative gene expression level was calculated using the 2^–ΔΔCt^ method. Each real-time PCR experiment contained three technical replicates.

## Results

### Effect of Different Fe(III)-EDTA Supply Levels on the Physiological Characteristics of Maize With Different Fe Efficiencies

**FIGURE 2:**
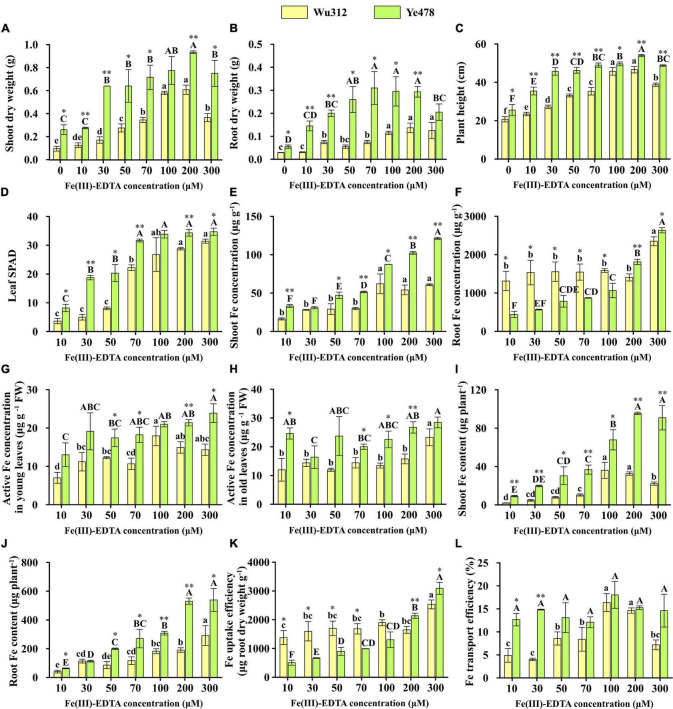
Shoot **(A)** and root dry weights **(B)**, Plant height **(C)**, SPAD value of the fourth leaf **(D)**, shoot **(E)** and root Fe concentrations **(F)**, active Fe concentration in young leaves **(G)** and old leaves **(H)**, shoot **(I)** and root Fe contents **(J)**, Fe uptake **(K)**, and transport **(L)** efficiencies of Fe-inefficient inbred line Wu312 and Fe-efficient inbred line Ye478 under different Fe(III)-EDTA levels in Experiment 1. Different lowercase and upper letters indicate significant difference (*p* < 0.05) of Wu312 and Ye478 among treatments, respectively. * and ** indicate significant difference between Wu312 and Ye478 at *p* < 0.05 and *p* < 0.01, respectively.

The shoot dry weight of Wu312 and Ye478 gradually increased with the increasing supply of Fe(III)-EDTA (Figure 2A). When the Fe supply level was 30 μM, Ye478 produced shoot dry weight like that on the supply of 100 μM. The shoot dry weight of two inbred lines reached the highest value when the Fe supply level was 200 μM. Nevertheless, when the Fe supply concentration was 300 μM, their plant growth was inhibited. Especially, the plant growth and development of Wu312 were significantly suppressed. Leaf SPAD of Ye478 and Wu312 increased with increasing Fe supply levels except for the leaf SPAD on the supply exceeding 100 μM (Figure 2D). In all treatments, leaf SPAD of Ye478 was significantly higher than that of Wu312 (Figure 2D). The leaf SPAD of Wu312 and Ye478 on the supply of 200 μM Fe(III)-EDTA was 5.8 and 1.8-fold higher than those on the supply of 30 μM, respectively.

**FIGURE 3:**
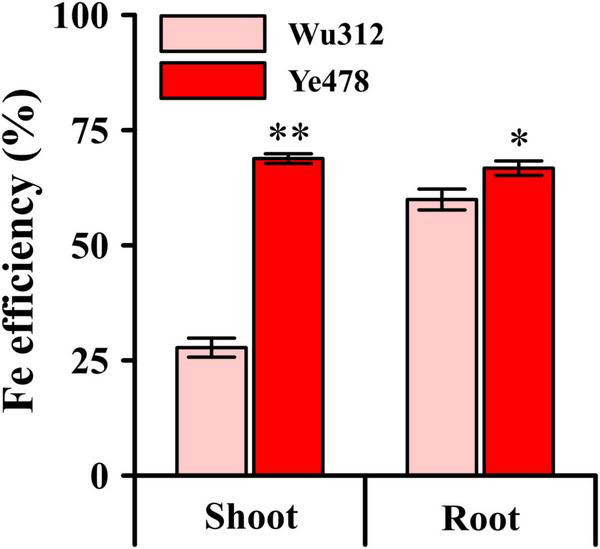
Iron (Fe) efficiency based on shoot and root dry weights. * and ** indicate significant difference between the Fe-inefficient (Wu312) and Fe-efficient (Ye478) inbred lines at *p* < 0.05 and *p* < 0.01, respectively.

Considering the dry matter weight, plant height, and leaf SPAD, as well as the observed plant growth in Experiment 1, this study determined the Fe supply level 200 μM as Fe-sufficient supply for normal growth. When the Fe supply level was 30 μM, the phenotypic difference between two inbred lines was the largest (Figures 2A–D). The shoot and root dry weights, plant height, and leaf SPAD of Ye478 were 3.8-, 2.7-, 1.7-, and 3.8-fold higher than those of Wu312 when the iron supply level was 30 μM Fe(III)-EDTA, respectively. Compared with the Fe-sufficient treatment, the shoot and root dry weights, plant height, and leaf SPAD of Ye478 on the supply of 30 μM Fe(III)-EDTA were decreased by 31.4, 31.8, 15.3 and 46.0%, respectively; while Wu312 was decreased by 72.0, 45.1, 41.6 and 84.1%, respectively. The R/S ratio of Ye478 showed no significant difference between the Fe-deficient treatment [30 μM Fe(III)-EDTA] and the Fe-sufficient treatment; while the R/S ratio of Wu312 on the supply of 30 μM Fe(III)-EDTA was the highest among all treatments (Supplementary Figure 1). In this study, Fe efficiency was calculated using the ratio of the value on the Fe-deficient supply of 30 μM to the value on the Fe-sufficient supply of 200 μM. As shown in Figure 3, the Fe efficiency based on the shoot and root dry weight of Ye478 was 68.8 and 66.7%, which was 2.5-fold and 1.1-fold significantly higher than those of Wu312, respectively.

The shoot Fe concentration of the two inbred lines showed an increasing trend as the Fe supply level increased (Figure 2E). Furthermore, the shoot Fe concentration of Ye478 was higher than that of Wu312. However, when the Fe supply levels ranged from 10 to 100 μM, the Fe concentrations in the roots of Wu312 were significantly higher than those of Ye478 (Figure 2F). In addition, a large amount of Fe might have accumulated in the root apoplast and root surface when only nitrate nitrogen was supplied, which may cause the root Fe concentration of two inbred lines to be too high. In both young and old leaves, the active Fe concentration of Ye478 was higher than that of Wu312 in most treatments (Figures 2G,H). However, there were no significant differences in active Fe concentration in young and old leaves of two inbred lines between the Fe-deficient and Fe-sufficient treatments. In the old leaves, the average active Fe concentrations of Ye478 and Wu312 were 23.2 and 15 μg g^–1^ FW, respectively. In the new leaves, the average active Fe concentration of Ye478 and Wu312 was 19.1 and 12.6 μg g^–1^ FW, respectively.

Fe contents in the shoots and roots of Wu312 and Ye478 displayed an increasing trend with the increase of Fe(III)-EDTA supply (Figures 2I,J). The shoot Fe contents of Wu312 and Ye478 on the supply of 200 μM were 7.5-fold and 3.4-fold higher than those under Fe-deficient supply (30 μM), respectively. Fe uptake efficiency of Ye478 increased with increasing Fe supply levels, and the uptake efficiency of Wu312 displayed no significant difference among the treatments except for 300 μM (Figure 2K). Moreover, the uptake efficiency of Wu312 on the supply of 30 μM was significantly higher than that of Ye478 under Fe-deficient supply (30 μM) and was markedly lower than that of Ye478 under Fe-sufficient supply (200 μM). The Fe transport efficiency of Wu312 displayed a significant reduction under the Fe-deficient condition when compared with the Fe-sufficient condition, and Ye478 displayed no significant difference between the two treatments (Figure 2L).

### Impact of Nitrogen Form on Maize With Different Iron Efficiencies Under Low Fe Stress

**FIGURE 4:**
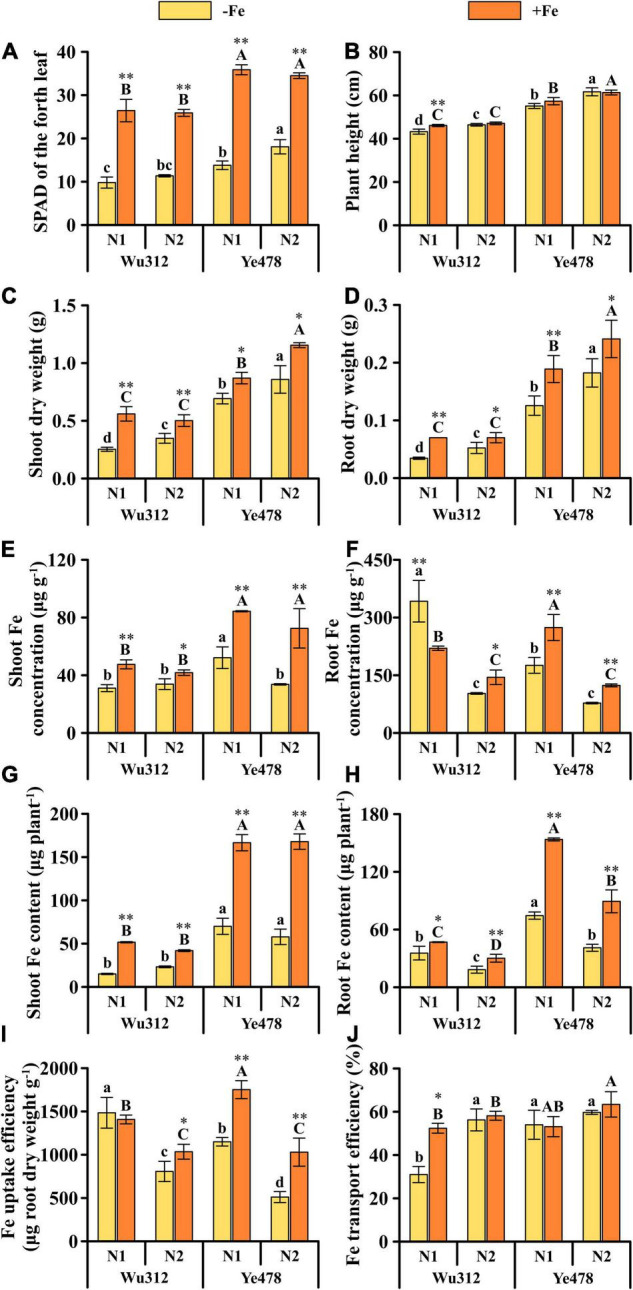
SPAD value of the fourth leaf **(A)**, plant height **(B)**, shoot **(C)** and root dry weights **(D)**, shoot **(E)** and root Fe concentrations **(F)**, shoot **(G)** and root Fe contents **(H)**, and Fe uptake **(I)** and transport efficiencies **(J)** of Wu312 and Ye478 under Fe-deficient [–Fe: 30 μM Fe(III)-EDTA] and Fe-sufficient conditions [+Fe: 200 μM Fe(III)-EDTA] supplied with N1 [Ca(NO_3_)_2_: 2 mM NO_3_^–^-N] or N2 [NH_4_NO_3_: 2 mM NO_3_^–^-N, 2 mM NH_4_^+^-N]. Different lowercase and upper letters indicate significant difference (*p* < 0.05) of inbred lines under –Fe and +Fe conditions, respectively. * and ** indicate significant difference between the –Fe and +Fe treatments at *p* < 0.05 and *p* < 0.01, respectively.

Under Fe deficiency, the SPAD for Ye478 in the N2 was 1.2-fold higher than that in the N1, whereas there was no significant difference in the SPAD of Wu312 between the N1 and N2 treatments (Figure 4A). Except for Wu312 under Fe-sufficient condition, shoot and root dry weights for two inbred lines supplied with nitrate-nitrogen (N1) were significantly lower than those simultaneously supplied with equivalent NH_4_^+^-N and NO_3_^–^-N(N2) (Figures 4C,D). Under low Fe stress, compared with N1, N2 increased the shoot and root dry weights of Wu312 by 37.5 and 51.6% and increased the shoot and root dry weights of Ye478 by 23.9 and 45.1%, respectively. Under Fe-sufficient condition, compared with N1, the shoot and root dry weights of Ye478 in the N2 treatment were increased by 32.8 and 27.6%, respectively. N2 treatment also increased the plant heights of two inbred lines except for the plant height of Wu312 under Fe-sufficient condition (Figure 4B). Under Fe-deficient condition, compared with N1, N2 reduced the shoot Fe concentration of Ye478 by 35.4% (Figure 4E).

The use of hydrochloric acid combined with a mixed cleaning solution of EDTA-Na and CaSO_4_ could significantly reduce the precipitation and fixation of Fe in the roots (Figures 2F, 4F). Compared with N1, N2 significantly reduced the root Fe concentration (Figure 4F). N2 decreased root Fe concentration of Wu312 under Fe-deficient and Fe-sufficient conditions by 70.0 and 34.3%, respectively; and reduced Ye478 by 55.8 and 54.9%, respectively. There were no significant differences in the shoot Fe contents of two inbred lines between the N1 and N2 treatments (Figure 4G). However, the root Fe contents of two inbred lines in the N1 treatment were higher than those in the N2 treatment (Figure 4H). Compared with the N1 treatment, N2 treatment decreased root Fe content of Wu312 in the −Fe and +Fe treatments by 48.5 and 35.6% and decreased Ye478 by 44.9 and 41.9%, respectively. Compared with N1, the Fe uptake efficiency of the two inbred lines also showed a significant reduction in the N2 treatment (Figure 4I). Under Fe-deficient conditions, the Fe uptake efficiencies of Wu312 and Ye478 were decreased by 45.6 and 55.5%, respectively. Also, compared with N1, the supply of equivalent NH_4_^+^-N and NO_3_^–^-N improved the Fe transport efficiency of Wu312 under Fe starvation (Figure 4J).

### Quantitative Trait Locus Analysis of Fe Deficiency Tolerance in Maize

#### Phenotypic Data Analysis

The Ye478 × Wu312 RIL population consisting of 218 lines was used to identify the loci associated with Fe deficiency tolerance in maize. In this study, phenotypic data of each trait under Fe-deficient condition (−Fe) and the ratios of the values under Fe-deficient condition to the values under Fe-sufficient condition (−Fe/+Fe) were used for QTL mapping. Here, the ratio of −Fe to +Fe for each trait was calculated using the BLUPs for each trait in the −Fe and +Fe treatment. The means and range of each trait for the parents and their RIL population are shown in Table 1. Our results indicated that Ye478 exhibited more tolerant to Fe deficiency when compared with Wu312. Fe-efficient inbred line Ye478 obtained higher leaf SPAD, plant height, shoot and root dry weights. Fe deficiency resulted in 64.5 and 74.2% decreases in leaf SPAD of Ye478 and Wu312, respectively. Fe deficiency led to a 66 and 75% reduction in leaf SPAD of Ye478 and Wu312, respectively. Plant heights of Ye478 and Wu312 under Fe deficiency were significantly decreased by 9 and 13%, respectively. Furthermore, Fe deficiency led to a 22 and 59% reduction in shoot dry weights of Ye478 and Wu312, respectively. Compared with Fe-sufficient condition, root dry weight of Ye478 under Fe-deficient condition was markedly decreased by 15%, and root dry weight of Wu312 was significantly reduced by 36%. Under Fe deficiency, the R/S ratio for Wu312 was markedly increased by 57%, whereas the R/S ratio of Ye478 displayed no significant difference between different treatments. Interestingly, the root length of Wu312 in the −Fe treatment was significantly higher than that of Ye478.

**TABLE 1:** Statistical analysis of phenotypic variation in parents and their recombinant inbred line (RIL) population.

Trait	Treatment	Parents		*a*	RIL population			
		Ye478	Wu312		Mean	Range	CV (%)	*H*^2^ (%)
SPAD	−Fe	11.6	6.9	**	9.3	2.8–20.3	39.8	57.8
	−Fe/+Fe	0.34	0.25	**	0.33	0.08–0.71	37.5	
PH	−Fe	51.2	41.6	**	48.9	33.3–70.3	12.9	83.3
	−Fe/+Fe	0.91	0.87	*	0.88	0.57–1.30	13.4	
RL	−Fe	41.5	47.0	*	48.5	12.3–74.1	17.9	85.8
	−Fe/+Fe	0.81	0.89	**	0.92	0.54–1.51	17.9	
SDW	−Fe	0.66	0.20	**	0.32	0.10–0.73	33.5	76.1
	−Fe/+Fe	0.78	0.41	**	0.63	0.24–1.49	34.1	
RDW	−Fe	0.22	0.09	**	0.11	0.05–0.23	30.5	83.2
	−Fe/+Fe	0.85	0.64	*	0.78	0.37–2.33	32.5	
R/S	−Fe	0.32	0.44	*	0.35	0.23–0.60	17.8	59.1
	−Fe/+Fe	1.03	1.57	**	1.38	0.81–2.70	19.8	

a * and ** indicate significant difference between Ye478 and Wu312 at p < 0.05 and p < 0.01, respectively. NS indicates no significant difference.

**FIGURE 5:**
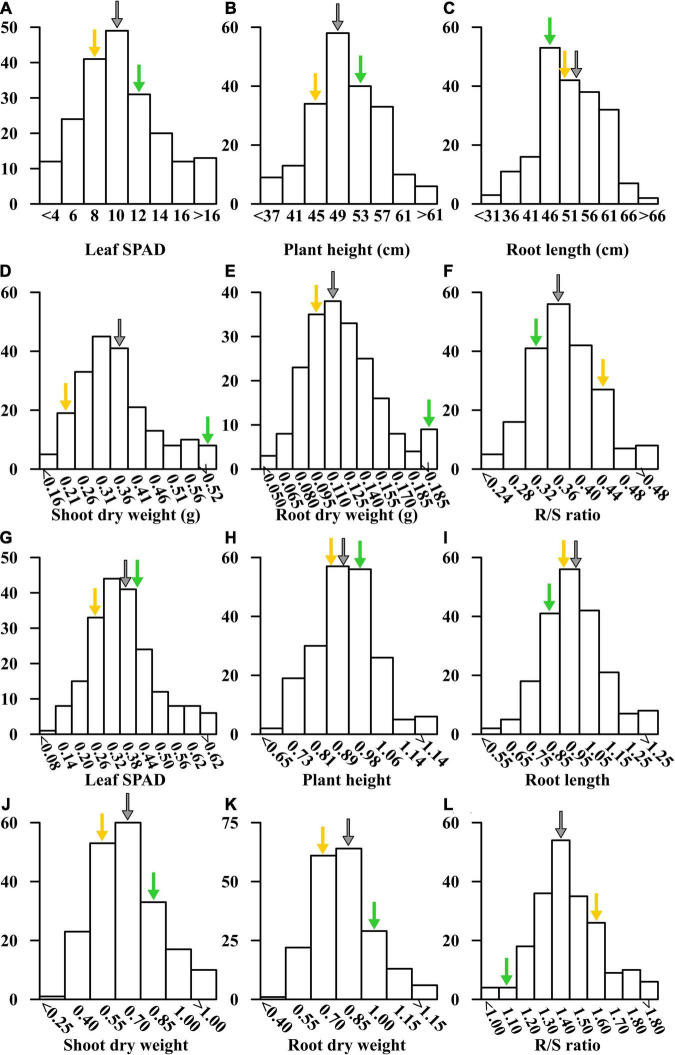
Distribution of SPAD value of the fourth leaf **(A,G)**, plant height **(B,H)**, root length **(C,I)**, shoot **(D,J)** and root dry weights **(E,K)**, and R/S ratio **(F,L)** in the –Fe and –Fe/+Fe treatments are shown in **(A–L)**, respectively. Yellow and green arrows represent the mean values for each trait of Fe-inefficient parent Wu312 and Fe-efficient parent Ye478, respectively. Gray arrows indicate the mean values of each trait in the Ye478 × Wu312 RIL population.

In the −Fe and −Fe/+Fe treatments, the means for leaf SPAD, plant height, shoot and root dry weights, R/S ratio were between the values of Fe-efficient inbred line Ye478 and Fe-inefficient inbred line Wu312 (Table 1 and Figure 5). The coefficient of variation (CV) for each trait under Fe-deficient nutritional status ranged from 12.9 to 39.8%, indicating large phenotypic variation among 218 RILs. Most traits displayed normal distributions based on the BLUPs for each trait among the RILs (Figure 5 and Supplementary Table 2).

Pearson’s correlation analysis among different traits in the −Fe treatment was performed, and the coefficients of correlation between each trait pair are shown in Table 2. There were positive significant correlations among plant height, root length, shoot and root dry weights under Fe-deficient nutritional status (*r* = 0.163–0.859, *p* < 0.05) (Table 2), indicating that inbred lines, which had higher plant height, may also have higher root length as well as more biomass accumulation.

**TABLE 2:** Pearson correlation coefficients between each trait under Fe deficiency.

Trait	SPAD *a*	PH	SDW	RDW	R/S
PH	0.146*				
SDW	0.339**	0.738**			
RDW	0.410**	0.650**	0.859**		
R/S	NS	−0.288**	−0.369**	NS	
RL	NS	0.244**	0.161*	0.278**	0.163*

a * and ** indicate significant difference at p < 0.05 and p < 0.01, respectively. NS indicates no significant difference.

#### Quantitative Trait Locus Detection

**FIGURE 6:**
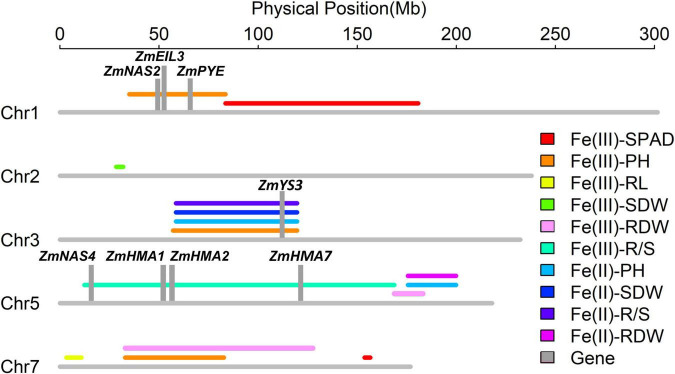
Ten quantitative trait loci associated with Fe deficiency tolerance detected on Chromosomes 1, 2, 3, 5, and 7 in the Ye478 × Wu312 recombinant inbred line (RIL) population and the co-localizations identified in the same population on the supply of Fe(III)-EDTA (Xu et al., 2022). Important candidate genes (*ZmYS3*, *ZmHMA1*, *ZmHMA2*, *ZmHMA7*, *ZmNAS2*, *ZmNAS4*, *ZmPYE*, and *ZmEIL3*) are depicted in gray columns. The lines of different colors indicate the QTLs controlling different traits, including leaf SPAD (SPAD), plant height (PH), root length (RL), shoot (SDW), and root (RDW) dry weights, and R/S ratio (R/S).

In this study, the BLUPs under Fe deficiency and the ratios of the BLUPs in the −Fe treatment to the values in the + Fe treatment for each trait were used to perform QTL analysis. Physical positions for each locus distributed detected in this study are shown in Figure 6. Details of each detected QTL, including QTL name, chromosomal position, 1-LOD marker interval, and physical interval, LOD value, additive effect, and percentages of total phenotypic variance explained (*R*^2^), are shown in Table 3. A total of ten quantitative trait loci controlling leaf SPAD, plant height, root length, shoot and root dry weights, and R/S ratio were identified on Chromosomes 1, 2, 3, 5, and 7, explaining 6.2–12.0% of phenotypic variation.

**TABLE 3:** Quantitative trait loci (QTLs) associated with Fe deficiency tolerance.

Trait	Treatment	Name	Chr	Marker interval (cM)	Physical interval (Mb) *a*	LOD	Additive effect *b*	R^2^ (%)	Reference
SPAD	−Fe	*qFe(III)-SPAD1-1*	1	umc2112–umc1754	84.4–184.8	2.9	1.55	6.2	Gu et al., 2015; Luo et al., 2017
		*qFe(III)-SPAD7-1*	7	bnlg1805–umc1710	162.6–165.5	3.4	−1.01	6.7	
PH	−Fe	*qFe(III)-PH1-1*	1	bnlg1484–umc2112	34.9–84.4	4.2	1.98	11.6	Zhang et al., 2018
	−Fe/+Fe	*qFe(III)-PH3-1*	3	umc1223–umc1773	57.5–120.9	3.1	−0.05	11.1	Hindu et al., 2018
		*qFe(III)-PH7-1*	7	phi034–umc1433	32.6–87.0	3.2	0.03	7.2	Benke et al., 2014
RL	−Fe	*qFe(III)-RL7-1*	7	umc1426–phi057	3.3–11.0	3.1	2.71	9.5	
SDW	−Fe	*qFe(III)-SDW2-1*	2	bnlg2248–umc2248	29.9–33.5	4.0	0.04	8.2	Gu et al., 2015; Zhang et al., 2018
RDW	−Fe	*qFe(III)-RDW5-1*	5	umc1221–bnlg278	172.0–186.9	3.3	−0.01	9.7	Benke et al., 2014, 2015; Luo et al., 2017
		*qFe(III)-RDW7-1*	7	phi034–mmc0411	32.6–133.1	3.3	0.01	6.3	Benke et al., 2014; Azevedo et al., 2015; Zhang et al., 2018
R/S	−Fe	*qFe(III)-R/S5-1*	5	phi113–umc1221	12.9–172.0	3.0	−0.02	12.0	Gu et al., 2015; Luo et al., 2017; Hindu et al., 2018

*a Physical interval accords B73_V5 reference.*

*b Positive values of additive effect indicate Ye478 alleles are in the direction of increase; negative values indicate Wu312 alleles are in the direction of increase.*

Two minor-effect QTLs controlling leaf SPAD were identified under Fe deficiency (Table 3). On Chromosome 1, *qFe(III)-SPAD1-1* was located in the interval of umc2112-umc1754, explaining 6.2% of phenotypic variation. Alleles from Ye478 increased leaf SPAD by 1.55 at this locus. *qFe(III)-SPAD7-1* was flanked by bnlg1805-umc1710 on Chromosome 7, accounting for 6.7% of phenotypic variation. Alleles from Wu312 contributed to increased leaf SPAD.

Three QTLs controlling plant height in the −Fe and −Fe/+Fe treatments and a single locus controlling root length under Fe-deficient condition were detected on Chromosomes 1, 3, and 7 (Table 3). The major-effect QTL *qFe(III)-PH1-1* detected under Fe deficiency was located in the interval of bnlg1484-umc2112 on Chromosome 1, explaining 11.6% of phenotypic variation. Alleles from Ye478 at this QTL increased plant height by 1.98 cm. Major-effect QTL *qFe(III)-PH3-1* and minor-effect QTL *qFe(III)-PH7-1* controlling plant height were identified on Chromosomes 3 and 7 in the −Fe/+Fe treatment, and accounted for 11.1 and 7.2% of phenotypic variation, respectively. A minor-effect QTL *qFe(III)-RL7-1* controlling root length was flanked by umc1426-phi057 on Chromosome 7. Alleles from Wu312 contributed to increased root length.

Four QTLs controlling shoot and root dry weights and the R/S ratio under Fe deficiency were identified on Chromosomes 2, 5, and 7, accounting for 6.3–12.0% of phenotypic variation. *qFe(III)-SDW2-1* controlling shoot dry weight was flanked by bnlg2248-umc2248 on Chromosome 2 and explained 8.2% of phenotypic variation. A favorable allele at this locus came from Fe-efficient parent Ye478. Two minor-effect QTLs [*qFe(III)-RDW5-1*, *qFe(III)-RDW7-1*] controlling root dry weight were identified on Chromosomes 5 and 7, explaining 9.7 and 6.3% of phenotypic variation, respectively. Major-effect QTL *qFe(III)-R/S5-1* was located in the interval of phi113-umc1221 on Chromosome 5, accounting for 12.0% of phenotypic variation.

### Quantitative Trait Locus Co-localization and Candidate Gene Identification

As shown in Figure 6, three QTL co-localization regions were identified on Chromosomes 3, 5, and 7. In this study, *qFe(III)-PH7-1* controlling plant height was co-localized with *qFe(III)-RDW7-1* for root dry weight on Chromosome 7. Besides, another two loci in this study were co-localized with the loci identified in the Ye478 × Wu312 RIL population on the supply of Fe(III)-EDTA (Xu et al., 2022). On Chromosome 3, *qFe(III)-PH3-1* detected on deficient supply of Fe(III)-EDTA in this study was co-localized with three loci [*qFe(II)-PH3-1*, *qFe(II)-SDW3-1*, and *qFe(II)-R/S3-1*] detected on the supply of ferrous iron. On Chromosome 5, *qFe(III)-RDW5-1* controlling root dry weight was co-localized with two QTLs [*qFe(II)-PH5-1* and *qFe(II)-RDW5-1*] mapped on the supply of ferrous iron.

Based on physical position, three QTL co-localization identified by nine QTLs for different traits on Chromosomes 3, 5 and 7 were selected for the identification of candidate genes. Three intervals were identified at 32.6–87.0 Mb on Chromosomes 7, 59.0–120.9 Mb on Chromosome 3, and 178.9–186.9 Mb on Chromosome 5. A total of 426 candidate genes were identified: 149 genes on Chromosome 7 (Supplementary Table 3), 165 genes on Chromosome 3 (Supplementary Table 4), and 112 genes on Chromosome 5 (Supplementary Table 5).

Based on the functional descriptions of 426 candidate genes in *Arabidopsis* and rice on MaizeGDB Database (see text footnote 1) and Gramene Database (see text footnote 2), three candidate genes were considered to be associated with Fe deficiency tolerance, including *ZmYS3* (GRMZM2G063306), *ZmEIL8* (GRMZM2G040481), and *ZmMYB153* (GRMZM2G050550) (Table 4). Beyond that, according to previous reports on Fe deficiency, ten genes associated with the tolerance to Fe deficiency were identified within three loci [*qFe(III)-PH1-1*, *qFe(III)-PH3-1*, and *qFe(III)-R/S5-1*], including *ZmNAS2* (GRMZM2G030036), *ZmNAS4* (GRMZM2G439195), *ZmEIL3* (GRMZM2G033570), *ZmPYE* (GRMZM2G350312), *ZmHMA1* (GRMZM2G067853), *ZmHMA2* (GRMZM2G099191), *ZmHMA7* (GRMZM2G029951), and *ZmILR*s (GRMZM5G 856837, GRMZM2G058451) (Table 4).

**TABLE 4:** Candidate genes associated with Fe deficiency tolerance in maize.

Chr	QTL	Gene ID	Physical position (bp) *a*	Description
1	*qFe(III)-PH1-1*	GRMZM2G030036	49167571–49169813	ZmNAS2 – Nicotianamine synthase 2
		GRMZM2G033570	52825403–52829476	ZmEIL3 – Ethylene insensitive-like 3
		GRMZM2G350312	65829055–65832129	ZmPYE – POPEYE (PYE), a bHLH transcription factor regulating response to iron deficiency
3	*qFe(III)-PH3-1*	GRMZM2G063306	99216253–99223276	ZmYS3 – Yellow stripe 3
5	*qFe(III)-R/S5-1*	GRMZM2G439195	16517168–16518643	ZmNAS4 – Nicotianamine synthase 4
		GRMZM2G069198	44649475–44655067	ZmNRAT1 – nramp aluminum transporter 1
		GRMZM2G067853	53832752–53854680	ZmHMA1 – Heavy metal ATPase 1
		GRMZM2G099191	58268972–58275897	ZmHMA2 – Heavy metal ATPase 2
		GRMZM5G856837	71442613–71445439	ZmILR3 – Transcription factor ILR3,bHLH-transcription factor 129
		GRMZM2G029951	124077732–124083350	ZmHMA7 – Heavy metal ATPase 7
		GRMZM2G058451	164356845–164360942	ZmILR3 – Transcription factor ILR3,bHLH-transcription factor 164
		GRMZM2G040481	179420089–179421819	ZmEIL8 – EIL-transcription factor 8
7	*qFe(III)-PH7-1*,	GRMZM2G050550	52720492–52722060	MYB153 – MYB-transcription factor 153
	*qFe(III)-RDW7-1*			

*a Physical position of genes accords B73_V5 reference.*

### Expression of Candidates Associated With Fe Deficiency Tolerance in Maize

**FIGURE 7:**
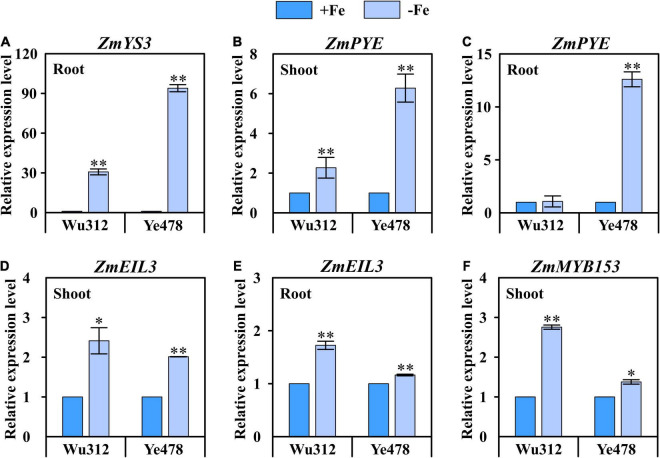
Expression of four candidate genes in the shoots and roots of Fe-inefficient (Wu312) and Fe-efficient (Ye478) parents under Fe-deficient [–Fe: 30 μM Fe(III)-EDTA] and Fe-sufficient [+Fe: 200 μM Fe(III)-EDTA] conditions. **(A)** *ZmYS3* expression in the roots, **(B,C)** *ZmPYE* and **(D,E)** *ZmEIL3* expression in the shoots and roots, **(F)** *ZmMYB153* expression in the shoots. * and ** indicate significant difference between the –Fe and +Fe treatments at *p* < 0.05 and *p* < 0.01, respectively.

According to the candidate genes identified in Experiment 3, the expression of *ZmYS3* (GRMZM2G063306), *ZmPYE* (GRMZM2G350312), *ZmEIL3* (GRMZM2G033570), and *ZmMYB153* (GRMZM2G050550) was assessed in Fe-deficient and Fe-sufficient shoots and roots of Fe-inefficient parent Wu312 and Fe-efficient parent Ye478 (Figure 7).

It should be noted that *ZmYS3*, which encodes a yellow-stripe protein, was only expressed in the roots of Wu312 and Ye478. For Fe-inefficient parent Wu312, *ZmYS3* was significantly (*p* < 0.01, 31-fold) upregulated under Fe deficiency. A 94-fold upregulation was observed in Fe-deficient roots of Fe-efficient parent Ye478, which was more than three times higher than that for Wu312 (Figure 7A). Interestingly, *ZmPYE*, a homolog of *POPEYE*, which plays a vital role in Fe homeostasis in strategy I plants in rice and non-gramineous plants, was significantly highly induced by Fe deficiency in the shoots and roots of two inbred lines. In the shoots, *ZmPYE* was 2-fold and 6-fold upregulated by Fe deficiency in Wu312 and Ye478, respectively (Figure 7B). In the roots, 13-fold upregulation induced by Fe deficiency was found in Ye478; however, no change in expression was observed in Wu312 (Figure 7C). Similar to *ZmYS3*, the upregulation of *ZmPYE* induced by low Fe stress was greater in Ye478 than in Wu312. These findings implicated that candidate genes *ZmYS1* and *ZmPYE* may be responsible for untangling genotypic differences in tolerance to Fe deficiency between the Fe-inefficient parent Wu312 and Fe-efficient parent Ye478.

The *ZmEIL3*, a homolog of *ETHYLENE INTENSITIVE3*, which is the key for ethylene regulation and essential for Fe homeostasis in *Arabidopsis*, was induced by low Fe stress in both shoots and roots of two inbred lines (Figures 7D,E). Moreover, *ZmMYB153* was only significantly upregulated in the shoots of two parents (Figure 7F). These results indicated that *ZmEIL3* and *ZmMYB153* may be involved in the mechanisms of Fe deficiency tolerance in maize.

## Discussion

### Physiological Evaluation of Fe Efficiency in Maize Seedlings Adapted to Low-Fe Stress

Previous studies have shown that Fe-efficient genotypes should not only be able to absorb more Fe from deficient soils but should also produce more dry matter and grain yield (Krishnasamy et al., 2005; Long et al., 2020), which was consistent with the results in the current study. On the supply of 30 μM Fe(III)-EDTA in Experiment 1, shoot and root dry weights of Fe-efficient Ye478 were 3.8-fold and 2.7-fold higher than those of Fe-inefficient Wu312, respectively (Figures 2A,B). Also, the Fe efficiencies based on the shoot and root dry weights of Ye478 were 68.8 and 66.7%, respectively, which were significantly higher than those of Wu312 (Figure 3). Fe is an essential component of several Fe-S proteins involved in the chloroplast electron transfer chain (Yousfi et al., 2009). The reason why the dry weight of Wu312 decreased significantly may be related to the alteration in chloroplasts, the corresponding inhibition of cell division, and the activation of several glycolytic enzymes (Rabotti et al., 1995; Espen et al., 2000). Rengel and Römheld (2000) demonstrated that the shoot dry weight and relative shoot growth were the most suitable parameters for distinguishing the ability of plants to tolerate Fe deficiency. Moreover, Fe deficiency response requires an overall increase in carbon import to the roots, favoring root expansion (Jin et al., 2008). Fe deficiency stress leads to an increase in the R/S ratio, indicating a less reduction of shoot growth when compared with root growth (Cianzio and Voss, 1994). In this study, compared with Fe-sufficient supply, we observed a significant increase of the R/S ratio in Fe-inefficient inbred line Wu312 and no significant difference of the R/S ratio in Fe-efficient inbred line Ye478 on the Fe supply of 30 μM (Figure 2C). This indicated that the R/S ratio displayed a markedly genotypic difference of the allocation of biomass in different organs between Fe-efficient and Fe-inefficient genotypes in response to low-Fe nutrient availability. Crowley et al. (2002) reported that Fe starvation altered R/S ratios in different barley cultivars and suggested this parameter as a criterion for selecting Fe-efficient plants. Phytosiderophores (PS) not only promote the root uptake of Fe but also regulate the translocation of Fe to the shoot as the Fe-PS complex (Nogiya et al., 2016). However, Zhang et al. (1991) reported a relatively poor relationship between PS exudation and Fe deficiency tolerance of barley genotypes. And, in several maize cultivars, Fe deficiency tolerance was reported not to be related to the total amounts of secreted PS (von Wirén et al., 1994; Yousfi et al., 2009).

Shoot Fe concentration of Ye478 was higher than that of Wu312 (Figure 2E). However, when the Fe supply level changed from 10 to 100 μM, the root Fe concentration of Wu312 was significantly higher than that of Ye478 (Figure 2F). The confirmed results from some cereal species indicated that the concentration of Fe is not a reliable indicator for distinguishing sensitivity to Fe deficiency among genotypes (Mohamed et al., 2003; Essa et al., 2015). Moreover, it has been established that, when plants are grown under Fe deficiency in field conditions, no significant correlation is found between Fe concentration and crop tolerance to Fe deficiency (Eshghizadeh et al., 2015). Iron chlorosis is due to chlorophyll dilution when leaves continue to grow at a normal rate under Fe deficiency (Abadía et al., 1999). The difference in the pigment dilution rates was directly related to the dissimilarity in growth rates (leaf expansion rate) (Yousfi et al., 2009). Combined with the results of visual observation and shoot dry weight, the growth rate of Ye478 plants was significantly higher than that of Wu312 (Figure 2A). Simultaneously, the SPAD value of Ye478 was also higher than that of Wu312 (Figure 2D). Therefore, the SPAD value is also a suitable index for evaluating Fe efficiency. An efficient root-to-shoot translocation of Fe, under Fe-deficient condition, has been causally related to the Fe deficiency tolerance in rice and wheat (Nogiya et al., 2016). However, our results showed that the Fe transport efficiency of two inbred lines did not display a significant difference in most treatments (Figure 2L). Taken together, shoot and root dry weights, R/S ratio, leaf SPAD, and plant height are suitable for evaluating the tolerance to Fe deficiency in maize.

### Effects of Different Nitrogen Forms on Maize Growth and Fe Nutritional Status

In this study, the nitrogen (N) supply form of Experiment 1 was to supply only nitrate-nitrogen recommended by Hoagland nutrient solution. The results showed that, when the Fe supply concentration of Wu312 was as high as 300 μM, it had an inhibitory effect on plant growth. There was still obvious inter-vein chlorosis. The roots of two inbred lines precipitated and fixed a lot of Fe. Because the Fe nutrition of plants can be significantly affected by the form of N supply through altering the rhizosphere pH, apoplastic pH, and the uptake ratio of cations and anions (Zou et al., 2001). For example, in sunflower, supply of NO_3_^–^-N increased leaf apoplastic pH and depressed Fe(III) reductase activity and hence induced Fe chlorosis (Kosegarten et al., 1999). However, in ryegrass, switching the root N source from nitrate to ammonium causes a concomitant decrease in apoplastic pH (Schjoerring et al., 2002). The Fe availability to plants could increase 1,000-fold with a decrease in pH, such as from pH 8 to pH 7 (Guo et al., 2014). This study used different nitrogen supply forms to improve the growth of plants under Fe starvation and, at the same time, reduce the accumulation of Fe in roots.

It has been reported that ammonium as the sole N source can inhibit plant growth, including maize seedlings (Wang et al., 2015; Esteban et al., 2016). However, ammonium nitrogen can improve plant tolerance to a variety of abiotic stresses and enhance resistance to pathogen infection (Hessini et al., 2013; Vega-Mas et al., 2017; Xun et al., 2020). In addition, nitrate may stimulate the xylem loading of ammonium and/or its assimilation through the enhanced biosynthesis of amino acids (Kiyomiya et al., 2001). However, co-provision of nitrate and ammonium is beneficial for plant growth beyond that observed with either N source provided alone (Carlisle et al., 2012; Luo et al., 2013). Like other major crops, maize plants achieve optimal growth and yield under mixed N nutrition of nitrate and ammonium (Gu et al., 2013; Hessini et al., 2017; Ravazzolo et al., 2020), which were consistent with our results. In Experiment 2 of this study, N2 significantly increased the shoot and root dry weights of Wu312 by 37.5 and 51.6% and enhanced the shoot and root dry weights of Ye478 by 23.9 and 45.1% under Fe-deficient condition, respectively (Figures 4C,D). Beyond that, under Fe-sufficient condition, the shoot and root dry weights of Ye478 were increased by 32.8 and 27.6%, respectively (Figures 4C,D). The application of ammonium stimulates lateral roots (LRs) branching, whereas nitrate stimulates LR elongation, suggesting that the application of nitrate and ammonium has local and complementary effects on LR development (Hachiya and Sakakibara, 2017). This may also improve the tolerance to low-Fe stress of the two inbred lines. The synergistic beneficial effects of co-provision mainly arise from the reciprocal influences between NH_4_^+^ and NO_3_^–^ on their uptake, root morphology, transport of N compounds from roots to shoots, and plant C metabolism (Prinsi and Espen, 2018).

In this study, compared with Experiment 1, root Fe concentration in Experiment 2 was substantially decreased, which may be mainly due to the cleaning of roots using a mixed solution of EDTA-Na and CaSO_4_ and the solution of HCl. In this research, the supply of ammonium nitrate (N2) markedly reduced the root Fe concentrations of Wu312 and Ye478 in the −Fe and +Fe treatments (Figure 4F). This could be that external Fe was immobilized in the root apoplast or onto the root surface due to high pH and typical nucleation of precipitated Fe (Strasser et al., 1999). However, co-provision of nitrate and ammonium could have caused dissolution or remobilization dissolution of the Fe by lowering pH, resulting in a relatively low concentration of Fe in the roots (Zou et al., 2001). Besides, N2 also significantly reduced the root Fe contents of these two inbred lines under Fe-deficient and Fe-sufficient conditions; however, such differences were not shown in shoot Fe contents (Figures 4G,H). This may be attributed to the reduction of Fe(III)-citrate that might be depressed by the high root apoplastic pH, disrupting Fe translocation to the shoots (Mengel et al., 1994).

### Comparison of Detected Loci With Previous Studies

Numerous studies have been related to the concentrations of Fe and other heavy metals in grains and seedling leaves (Blair et al., 2010a; Mamo et al., 2014; Zdunić et al., 2014; Gu et al., 2015; Hindu et al., 2018; Izquierdo et al., 2018; Jeong et al., 2020). Nevertheless, there are very few previous studies focusing on Fe deficiency tolerance in crops (Blair et al., 2010b; Peiffer et al., 2012), and only a few researchers have reported the loci associated with the performance under Fe deficiency in maize (Benke et al., 2014, 2015).

In total, eight of ten QTLs detected in this study were co-localized with the loci controlling different traits reported in other pieces of research (Table 3). The *qFe(III)SPAD1-1*, *qFe(III)-SDW2-1*, *qFe(III)PH3-1*, and *qFe(III)-RDW5-1* identified in this study were co-localized with the loci controlling Zn and manganese (Mn) concentration in grains (Gu et al., 2015; Hindu et al., 2018), indicating that these QTL regions may have pleiotropic effects on mineral concentration of grains in maize. Intervals of *qFe(III)SPAD1-1*, *qFe(III)-R/S5-1*, and *qFe(III)-RDW5-1* were overlapped by the genomic regions identified by the loci controlling plant height under salt stress detected by Luo et al. (2017), which suggested that these QTL regions may have pleiotropic effects on plant height under abiotic stress. Two QTLs [*qFe(III)-R/S5-1* and *qFe(III)-RDW5-1*] controlling root dry weight under Fe deficiency in this work were co-localized with the loci controlling the same trait under Fe-deficient condition identified by linkage and association analysis (Benke et al., 2014, 2015). Besides, intervals of these two loci were also overlapped by the genomic regions of the QTLs controlling plant height under normal and external ethylene conditions, root diameter under low-P stress, and SPAD under low-Fe stress (Benke et al., 2014; Azevedo et al., 2015; Luo et al., 2017; Zhang et al., 2018). This implicated that these gnomic regions may harbor several genes with pleiotropic effect not only on root biomass accumulation but also on plant height, root morphological traits, and leaf SPAD at the seedling stage.

### Candidate Genes Associated With Fe Deficiency Tolerance

The GRMZM2G063306, also known as *ZmYS3*, was detected in the QTL co-localization of *qFe(III)-PH3-1*, *qFe(II)-PH3-1*, *qFe(II)-SDW3-1* and *qFe(II)-R/S3-1*, which were identified on the supply of Fe(II) and Fe(III). It is reported that *ZmYS3* was substantially induced by Fe deficiency in maize roots (Nozoye et al., 2013). Fe deficiency-inducible genes, such as *ZmTOM1*, *ZmDMAS1* and *ZmIRT1*, are upregulated in the roots of the *ys3* mutant, even under Fe-sufficient condition, suggesting that *ys1* and *ys3* plants are Fe deficient during growth in presence of Fe (Nozoye et al., 2013, 2015). Unspliced introns of *ZmTOM1* were detected only in *ys3*, rather than in *YS1YS3* or *ys1*, suggesting that *ZmTOM1* may be involved in the *ys3* phenotype (Nozoye et al., 2013, 2015). Furthermore, our results indicated that *ZmYS3* was remarkably upregulated in the roots of Wu312 and Ye478 under Fe deficiency, and the upregulation was greater in the Fe-efficient genotype than in the Fe-inefficient genotype (Figure 7A). These findings indicated that *ZmYS3* is involved in strategy II Fe acquisition and may play important roles in differing the ability to tolerate low-Fe stress in maize.

Apart from *ZmYS3*, other candidate genes may be involved in Fe homeostasis in strategy I plants and heavy metals transport in plants, which may form a sophisticated network underlying Fe deficiency tolerance. The GRMZM2G350312 identified within *qFe(III)-PH1-1* under deficient supply of Fe(III) in this study, encodes a bHLH transcription factor regulating the response to Fe deficiency and is identified to be *POPEYE* (*PYE*) in maize. The POPEYE (PYE belongs to IVb subgroup bHLHs) functions as an important repressor in the Fe deficiency regulatory network (Wang et al., 2018; Cheng et al., 2020). The PYE targets key genes involved in metal ion homeostasis, including a Zn transport facilitator (*ZIF1*), a plasma membrane-localized ferric chelate reductase from the same family as FRO2 (*FRO3*), and a nicotianamine synthase (*NAS4*) (Dinneny et al., 2008; Jeong et al., 2008; Curie et al., 2009). The PYE protein is localized in the nuclei of all cells within Fe-deficient *Arabidopsis* roots and is most highly expressed in the pericycle within the maturation zone (Long et al., 2010). The *pye-1* mutant exhibits chlorosis and root growth inhibition under Fe deficiency, which is associated with decreased elongation and swelling of root cells (Hindt and Guerinot, 2012; Kobayashi and Nishizawa, 2012). Additionally, in *pye-1*, several other Fe homeostasis genes are upregulated, including *OPT3*, *FRD3*, *NRAMP4*, *bHLH39* and *bHLH101* (Green and Rogers, 2004; Stacey et al., 2008; Long et al., 2010). In this research, we found that *ZmPYE* was not only significantly upregulated in the roots but also in shoots under Fe deficiency, and a greater upregulation was observed in Fe-efficient maize inbred line Ye478 (Figures 7B,C). This indicates that *ZmPYE*, which is involved in Fe homeostasis in strategy I plants, may play a vital role in Fe efficiency in maize.

The GRMZM2G033570, also known as *EIL3* encoding ETHYLENE INSENSITIVE3-LIKE1 protein in maize, was identified within *qFe(III)-PH1-1* under Fe-deficient condition. Besides a potential role in ethylene signaling, EIL3 (also called SLIM1 for SULFUR LIMITATION1), which participates in the modulation of S-deficiency response, is the only identified transcription factor in plant S-metabolism (Van der Ent et al., 2008; Filiz et al., 2017). The EIL3 function as a central transcriptional regulator, which control both the activation of sulfate acquisition and degradation of glucosinolates under –S conditions (Maruyama-Nakashita et al., 2006). It is reported that MYB72 physically interacts *in vitro* with the EIL3, which is induced under Fe deficiency (Van der Ent et al., 2008; García et al., 2011). This tandem MYB72-EIL3 may play a role in the regulation of Fe-deficiency responses (García et al., 2010).

In addition, GRMZM2G050550 that encodes MYB-transcription factor 153 was detected within *qFe(III)-PH7-1* in the −Fe/+Fe treatment. MYB transcription factors play pivotal roles in hormone conduction signaling and abiotic stress response (Zhang et al., 2021). Some members of the MYB family (members of the myeloblastosis family), which may be implicated in the PYE-mediated Fe deficiency response, have been implicated in Fe redistribution through the regulation of NAS4 (Palmer et al., 2013; Brumbarova et al., 2015). The TPL/TPR transcriptional co-repressors play very important roles in various phytohormone signaling pathways (including jasmonic acid and strigolactone). Some studies speculate that ZmMYB153 recruits the LisH or CTLH domains of some proteins in the TPL/TPRs family through its LSLSL-type EAR motif to form a complex, thereby inhibiting the expression of the relevant genes (Bai et al., 2020). This affects how maize responds to biotic and abiotic stresses.

Our results indicated that *ZmEIL3* was significantly upregulated in the Fe-deficient shoots and roots of maize inbred lines (Figures 7D,E), and *ZmMYB153* was Fe-deficiency inducible in maize roots (Figure 7F), further indicating that *ZmEIL3* and *ZmMYB153* may be involved in Fe deficiency tolerance in maize, which is considered as a strategy II plant.

## Data Availability Statement

The original contributions presented in the study are included in the article/Supplementary Material, further inquiries can be directed to the corresponding author/s.

## Author Contributions

JX, FYa, and XZhu performed the experiments. JX and XZhu analyzed the data. JX, XZhu, FYa, and HZ wrote the manuscript. FYu designed the study and modified the manuscript. All authors contributed to the article and approved the submitted version.

## Conflict of Interest

The authors declare that the research was conducted in the absence of any commercial or financial relationships that could be construed as a potential conflict of interest.

## Publisher’s Note

All claims expressed in this article are solely those of the authors and do not necessarily represent those of their affiliated organizations, or those of the publisher, the editors and the reviewers. Any product that may be evaluated in this article, or claim that may be made by its manufacturer, is not guaranteed or endorsed by the publisher.

## Footnotes

1
http://www.maizeGDB.org

2
https://www.gramene.org
