# Supplementary material for: Identification of Quantitative Trait Loci Associated With Iron Deficiency Tolerance in Maize
Source: Front Plant Sci. 2022 Apr 14;13:805247. doi: 10.3389/fpls.2022.805247 (PMC9048261; doi:10.3389/fpls.2022.805247)
Supplement: Supplementary file 1 [file Data_Sheet_1.docx]

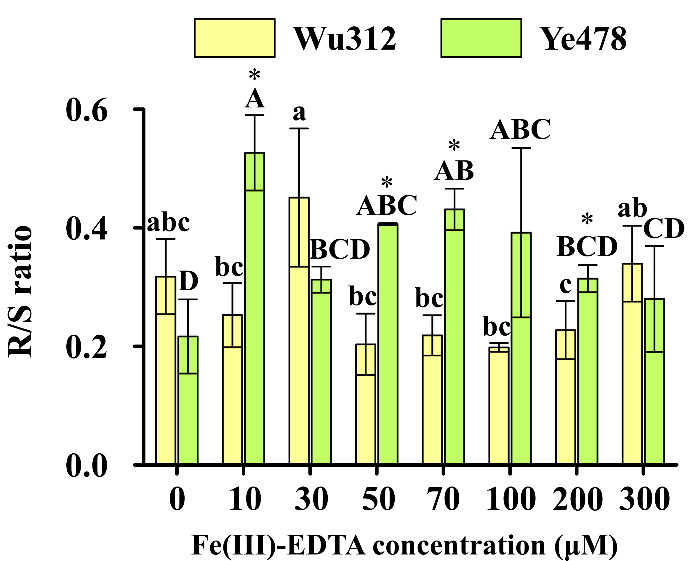


**FIGURE S1** R/S ratio of Fe-inefficient inbred line Wu312 and Fe-efficient inbred line Ye478 under different Fe(III)-EDTA levels in Experiment 1. Different lowercase and upper letters indicate significant difference (*P* < 0.05) of Wu312 and Ye478 among treatments, respectively. * indicates significant difference between Wu312 and Ye478 at *P* < 0.05.
